# Cytotoxic Effect of Vanicosides A and B from *Reynoutria sachalinensis* against Melanotic and Amelanotic Melanoma Cell Lines and in silico Evaluation for Inhibition of BRAFV600E and MEK1

**DOI:** 10.3390/ijms21134611

**Published:** 2020-06-29

**Authors:** Izabela Nawrot-Hadzik, Anna Choromańska, Renata Abel, Robert Preissner, Jolanta Saczko, Adam Matkowski, Jakub Hadzik

**Affiliations:** 1 Department of Pharmaceutical Biology and Botany, Wroclaw Medical University, 50556 Wroclaw, Poland; izabela.nawrot-hadzik@umed.wroc.pl; 2 Department of Molecular and Cellular Biology, Faculty of Pharmacy, Wroclaw Medical University, 50556 Wroclaw, Poland; anna.choromanska@umed.wroc.pl (A.C.); jolanta.saczko@umed.wroc.pl (J.S.); 3 Structural Bioinformatics Group, Institute for Physiology, Charité–University Medicine Berlin, 10115 Berlin, Germany; renata.abel@umed.wroc.pl (R.A.); robert.preissner@charite.de (R.P.); 4 Department of Dental Surgery, Wroclaw Medical University, 50425 Wroclaw, Poland; jakub.hadzik@umed.wroc.pl

**Keywords:** hydroxycinnamic esters, giant knotweed, melanoma, cell death, cytotoxicity, kinases

## Abstract

Vanicosides A and B are the esters of hydroxycinnamic acids with sucrose, occurring in a few plant species from the Polygonaceae family. So far, vanicosides A and B have not been evaluated for anticancer activity against human malignant melanoma. In this study, we tested these two natural products, isolated from *Reynoutria sachalinensis* rhizomes, against two human melanoma cell lines (amelanotic C32 cell line and melanotic A375 cell line, both bearing endogenous BRAFV600E mutation) and two normal human cell lines—keratinocytes (HaCaT) and the primary fibroblast line. Additionally, a molecular docking of vanicoside A and vanicoside B with selected targets involved in melanoma progression was performed. Cell viability was studied using an MTT assay. A RealTime-Glo™ Annexin V Apoptosis and Necrosis assay was used for monitoring programmed cell death (PCD). Vanicoside A demonstrated strong cytotoxicity against the amelanotic C32 cell line (viability of the C32 cell line was decreased to 55% after 72 h incubation with 5.0 µM of vanicoside A), significantly stronger than vanicoside B. This stronger cytotoxic activity can be attributed to an additional acetyl group in vanicoside A. No significant differences in the cytotoxicity of vanicosides were observed against the less sensitive A375 cell line. Moreover, vanicosides caused the death of melanoma cells at concentrations from 2.5 to 50 µM, without harming the primary fibroblast line. The keratinocyte cell line (HaCaT) was more sensitive to vanicosides than fibroblasts, showing a clear decrease in viability after incubation with 25 µM of vanicoside A as well as a significant phosphatidylserine (PS) exposure, but without a measurable cell death-associated fluorescence. Vanicosides induced an apoptotic death pathway in melanoma cell lines, but because of the initial loss of cell membrane integrity, an additional cell death mechanism might be involved like permeability transition pore (PTP)-mediated necrosis that needs to be explored in the future. Molecular docking indicated that both compounds bind to the active site of the BRAFV600E kinase and MEK-1 kinase; further experiments on their specific inhibitory activity of these targets should be considered.

## 1. Introduction

Melanoma is a malignant melanocytic tumor. The incidence of melanoma in the human population is ever-increasing and mortality in the group of patients with an advanced form of this disease is very high. Although melanoma accounts for only 4% of all skin cancers, it is responsible for >80% of skin cancer-related deaths [1]. The early-stage localized melanoma is treated with surgical interventions followed by chemotherapy and radiotherapy, but most patients are diagnosed at the time when the tumor has already metastasized—in such cases existing standard treatment methods show limited effectiveness [2]. A rarely occurring type of melanoma—amelanotic melanoma—which shows no or little pigmentation, is even more difficult to diagnose and is associated with a higher risk of death and recurrence [3]. In the last few years, many efforts were taken to improve understanding the features of melanoma cells. These have resulted in the approval by the Food and Drug Administration (FDA) of many new treatment methods directed towards the mitogen-activated protein kinase (MAPK) pathway which is ubiquitously activated in cutaneous melanoma. They include: BRAF inhibitors such as vemurafenib and dabrafenib used alone or in combination with the mitogen-activated protein kinase (MEK) inhibitor like trametinib dedicated to patients with a BRAFV600 mutation (35–50% of melanoma) [4]. This treatment reduces the tumor burden in almost all patients with a BRAFV600 mutation, but ultimately most patients will develop resistance [5]. Moreover, several immune therapies have also been approved for patients with metastatic melanoma, but their response rates are lower than those achieved with the targeted therapies [4]. In recent years, the significance of metabolic reprogramming in cancer initiation, maintenance, progression and development of chemoresistance has been often underlined [1,6].

Among natural products of plant origin, polyphenols have been found to target different hallmarks of cancer cells, often interfering with several of them at once, such as promoting apoptosis, regulating autophagy and inhibiting proliferation, and as well, they modulate the metabolic properties of cancer cells and the affect tumor-induced immunosuppressive behavior through the modulation of T-regulatory (Treg) cell populations and many others, including epigenetic modulation [7,8]. The natural plant compounds have also proved to be effective in repressing cancer chemoresistance [8]. Furthermore, different concentrations of polyphenols used against cancer cells may lead to various mechanisms of cancer cell suppression. For instance, the polyphenol diosmin induces apoptosis at high concentrations (20 μM), while at low concentrations (5 and 10 μM), it induces senescence in breast cancer cells [9].

In this study, we tested two rare polyphenols—vanicoside A and vanicoside B—which were isolated from rhizomes of *Reynoutria sachalinensis* [F.Schmidt] Nakai (giant knotweed, family Polygonaceae). This plant is native to East Asia but widely spread throughout Europe and North America becoming a noxious invasive pest. It is used traditionally as an herbal medicine in its natural distribution area for the treatment of arthralgia, jaundice, amenorrhea, cough, scalds and burns, traumatic injuries, carbuncles and sores [10]. Our earlier study showed that rhizomes of *R. sachalinensis* are a rich source of phenylpropanoid disaccharide esters with a predominant amount of vanicoside B [11,12,13]. The cytotoxicity of vanicosides A and B against melanoma cell lines has not been studied so far, but the cytotoxicity of other compounds belonging to disaccharide esters of phenylpropanoids was reported against different cancer cell lines [14,15]. So far, both vanicosides have been reported to act as PKC inhibitors and showed cytotoxicity against the MCF cell line at submicromolar dose levels [16]. Additionally, vanicoside B inhibited two-stage carcinogenesis of mouse skin tumor induced by 12-O-tetradecanoylphorbol-13-acetate (TPA) and exhibited antitumor activity against a panel of cancer cell lines in triple-negative breast cancer (TNBC) MDA-MB-231 cells by targeting cyclin-dependent kinase 8 (CDK8) [17].

**Figure 1:**
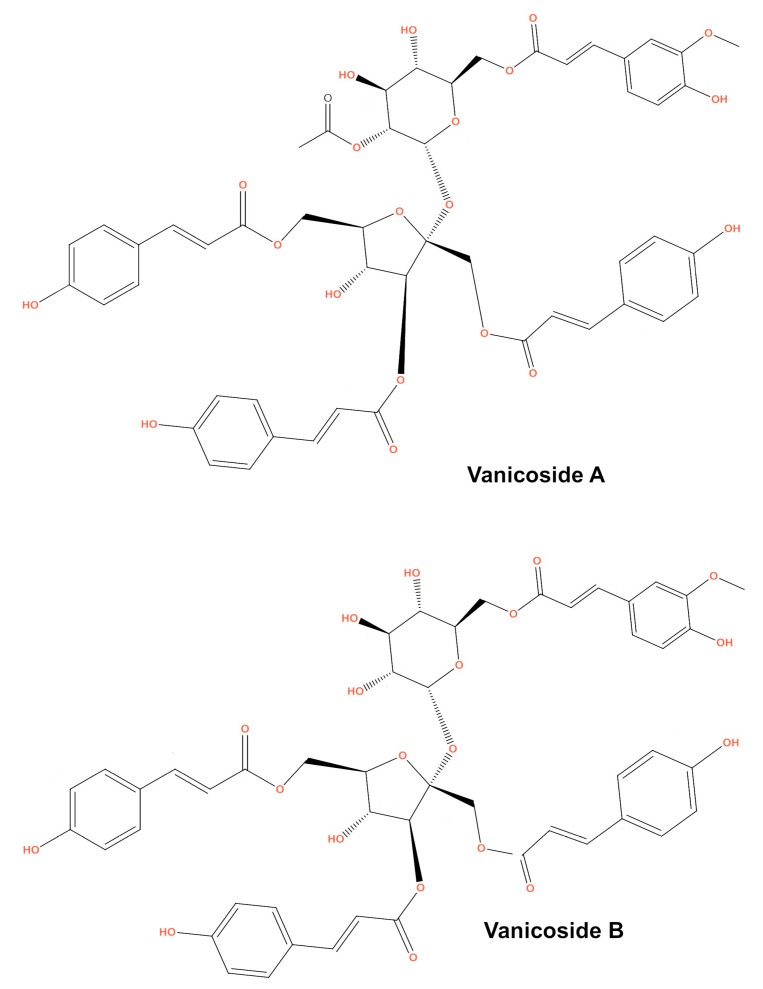
Chemical structure of vanicosides **A** and **B**.

This study was designed to investigate the anticancer effect of vanicosides A and B (Figure 1) against two melanoma cell lines: amelanotic malignant melanoma C32 (BRAFV600E) and malignant melanoma A375 (BRAFV600E). The cytotoxicity of vanicosides against two normal cell lines—keratinocytes (HaCaT) and the primary fibroblast line—was also tested. According to previous data, the hyperactivation of the ERK pathway by the BRAFV600E mutation in A375 cells is the main factor of its tumorigenesis [18]. The C32 cell line is also a BRAFV600E mutant [19]. Hence, we chose the A375 and C32 melanoma cell lines as a model to investigate the potential inhibition of BRAF-related molecular phenomena. Extensive literature concerning the cellular pathways implicated in melanomagenesis, ranging from signal transduction to developmental and transcriptional pathways and cell cycle deregulation is already available. Of the wide selection of therapeutic goals, our first study of vanicoside A and B activity against melanoma focused on the V600E BRAF and MEK1 molecules taking into account the big impact of potential inhibitors of these kinase on melanoma therapy and also other cancers like colon adenocarcinoma, papillary thyroid carcinoma and others [20]. Moreover, the V600E BRAF mutation concerns a number of patients, about 50% of melanomas of all clinical types have mutations in the BRAF kinase, mainly the V600E BRAF mutation (70%–80% of all BRAF mutations in all cancers; the remaining 5% to 15% are BRAF V600K, V600D or V600R mutations) [21]. The V600E BRAF mutation is also associated with more aggressive tumor features and reduced survival in melanoma patients [22]. Earlier studies on selective BRAF inhibitors proved high efficacy with acceptable side effects in patients with this mutation. Unfortunately, after the initial response, the development of acquired resistance occurs. Therefore, the search for new BRAF inhibitors becomes necessary. The main mechanisms of resistance involve the reactivation of ERK signaling [20], so the blocking of another step on the MAPK cascade using MEK inhibitors is being investigated. To learn whether vanicoside A and B possess a potential to interact with the BRAF(V600E) and MEK1 kinases, we performed molecular docking of vanicosides to the BRAF(V600E) and MEK1 proteins.

## 2. Results

### 2.1. Cell Viability–MTT Assay

**Figure 2:**
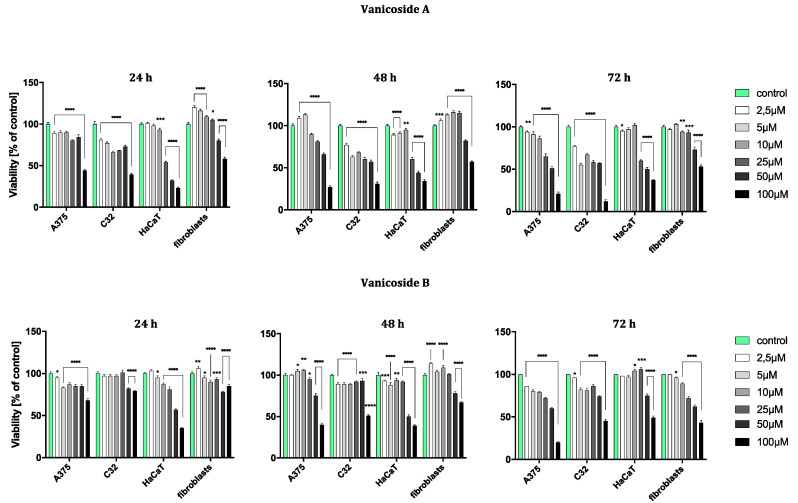
Viability of A375, C32, HaCaT and fibroblast cell line after 24, 48 and 72 h incubation following increasing concentrations of vanicoside A and vanicoside B. The mitochondrial metabolic function (viability) was expressed as a percentage of viable treated cells in relation to untreated control cells. Error bars shown in this figure are means ± SD for *n* ≥ 3. * Statistically significant at *p* ≤ 0.05, ** for *p* ≤ 0.01, *** for *p* ≤ 0.001, **** for *p* ≤ 0.0001. Formazan product from an MTT assay was not detectable for staurosporine (positive control) in any tested cell line.

The results obtained from the viability assay showed a decrease in survival of melanoma cells (Figure 2).

The amelanotic malignant melanoma C32 cell line was more sensitive to vanicoside A than other lines. The cytotoxicity of vanicoside A towards C32 cell line was observed beginning from the lowest tested concentration, 2.5 µM (81%, 77% and 77% cell viability for 24, 48 and 72 h, respectively), and increased with the concentration of vanicoside A and incubation time. Interestingly, treatment of the C32 cell line with doses between 5–50 µM of vanicoside A decreases cell viability to some similar level. The viability of the C32 cell line was decreased to 55% after 72 h incubation with 5.0 µM of vanicoside A. Another considerable decrease in cell viability was observed at the highest concentration of vanicoside A, 100 µM, for all incubation times (39%, 31% and 12% cell viability for 24, 48 and 72 h, respectively).

The malignant melanoma A375 cell line was less sensitive to vanicoside A than the amelanotic malignant melanoma C32 cell line. The viability of the A375 cell line slightly decreased after 24 h incubation with low concentrations (2.5, 5.0 µM) of vanicoside A, but then increased after 48 h incubation (113% cell viability at 5.0 µM). The toxic effect of vanicoside A started manifesting at 10 µM (90% cell viability) and was similar (not statistically significant for *p* ≤ 0.05) at all incubation times. A considerable decrease in viability of the A375 cell line (51% cell viability) was observed after 72 h incubation with 50.0 µM of vanicoside A. The highest tested concentration of vanicoside A (100 µM) caused a strong cytotoxic effect towards the A375 (44%, 27% and 21% cell viability for 24, 48 and 72 h, respectively) cell line.

The toxic effect of vanicoside A against keratinocytes (HaCaT) manifested at 25 µM and increased with the concentration of vanicoside A, but not with incubation time (54%, 60% and 60% cell viability for 24, 48 and 72 h, respectively). Cell viability slightly decreased after 24 and 48h incubation with low concentrations of vanicoside A (2.5–10.0 µM), but then, after 72h, increased to 102% (10.0 µM).

Fibroblasts appeared to be the least sensitive for vanicoside A among the tested cell lines. Fibroblast viability was severely affected only at higher concentrations of vanicoside A, 50 and 100 µM (80% and 58% cell viability, respectively, without significant difference among incubation times). Moreover, cell viability increased when fibroblasts were exposed to vanicoside A at low doses (2.5–25 µM) for 24 and 48 h. After 24 h, the highest cell viability (120%) was observed following exposure to 2.5 µM, and after 48h following exposure to 10 µM (116% cell viability).

Vanicoside B caused a weaker cytotoxic effect against the amelanotic malignant melanoma C32 cell line than vanicoside A. After 24h incubation, the viability of the C32 cell line was not decreased until using 50 and 100 µM of vanicoside B (approximately 80% cell viability). Longer treatment of the C32 cell line with doses up to 50 µM of vanicoside B slightly decreased cell viability to some similar level and a significant decrease was only noted when using the highest dose—100 µM (reduced cell viability by approximately 50% after 48 and 72 h).

The cytotoxicity of vanicoside B against the malignant melanoma A375 cell line was very similar to that of vanicoside A. A decrease in the cell viability of the A375 cell line after 24 h of incubation and then an increase after 48h incubation (with low doses 2.5–25 µM) were observed. After 72 h incubation, a decrease in cell viability was noted through all tested concentrations, the most significant one for the highest tested concentration—100 µM.

The cytotoxicity of vanicoside B was weaker compared with that of vanicoside A against keratinocytes (HaCaT). The viability of keratinocytes slightly decreased after 24 and 48 h incubation with low concentrations (2.5–25 µM) of vanicoside B, but then increased after 72h incubation (106% cell viability at 25 µM). After 72 h incubation, a decrease in cell viability was not noted until using 50 and 100 µM of vanicoside B.

The fibroblasts appeared to be similarly low-sensitive for vanicosides B and A, with little differences in the increase in cell viability after using lower concentrations of vanicoside B. Moreover, after 72 h incubation, the cytotoxicity of vanicoside B was manifested at a lower concentration, 25 µM (72% cell viability), than vanicoside A.

### 2.2. RealTime-Glo™ Annexin V Apoptosis and Necrosis Assay

**Figure 3:**
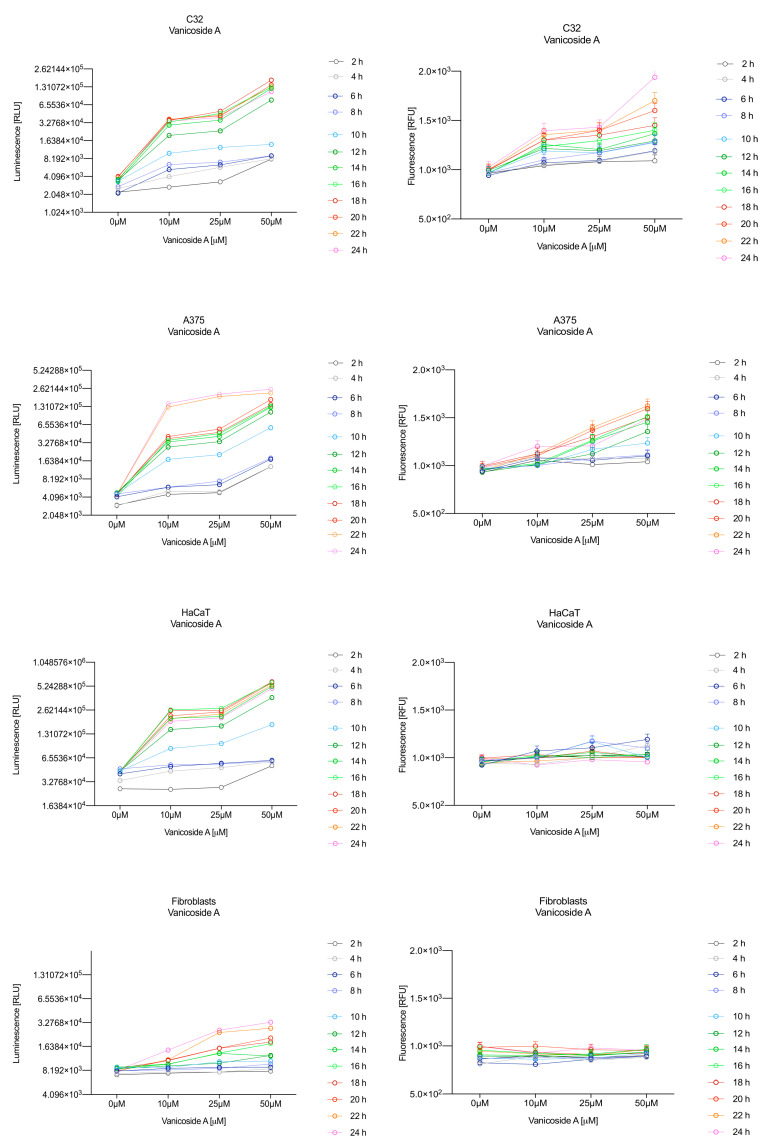
Repeated measurements of luminescence and fluorescence signals. C32, A375, HaCaT and fibroblast cell lines were exposed to serial dilutions of vanicoside A in the presence of RealTime-GloTM Annexin V Apoptosis and Necrosis Assay reagent. Relative luminescence units (RLU) (left panels, phosphatidylserine (PS): annexin V binding) and relative fluorescence units (RFU) (right panels; membrane integrity) were collected at indicated times. Data represent the mean of 3 readings for each replicate ± SD.

**Figure 4:**
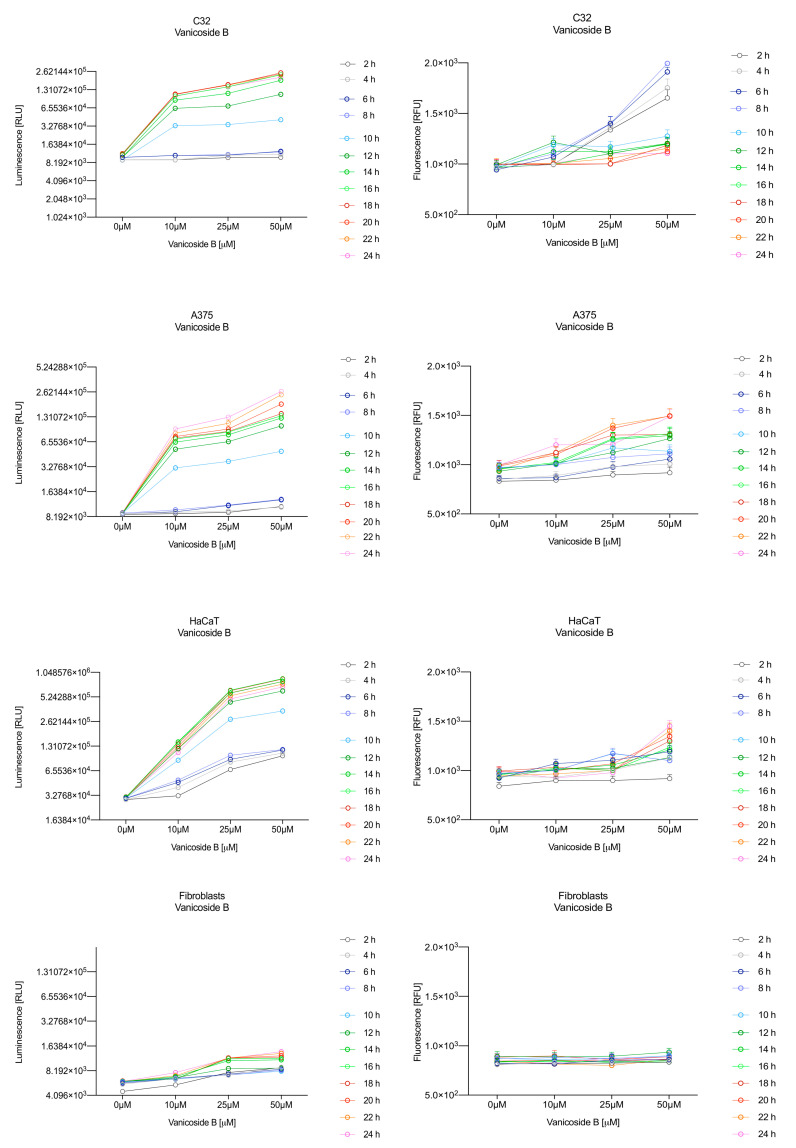
Repeated measurements of luminescence and fluorescence signals. C32, A375, HaCaT and fibroblast cell lines were exposed to serial dilutions of vanicoside B in the presence of RealTime-GloTM Annexin V Apoptosis and Necrosis Assay reagent. RLU (left panels, PS: annexin V binding) and RFU (right panels; membrane integrity) were collected at indicated times. Data represent the mean of 3 readings for each replicate ± SD.

**Figure 5:**
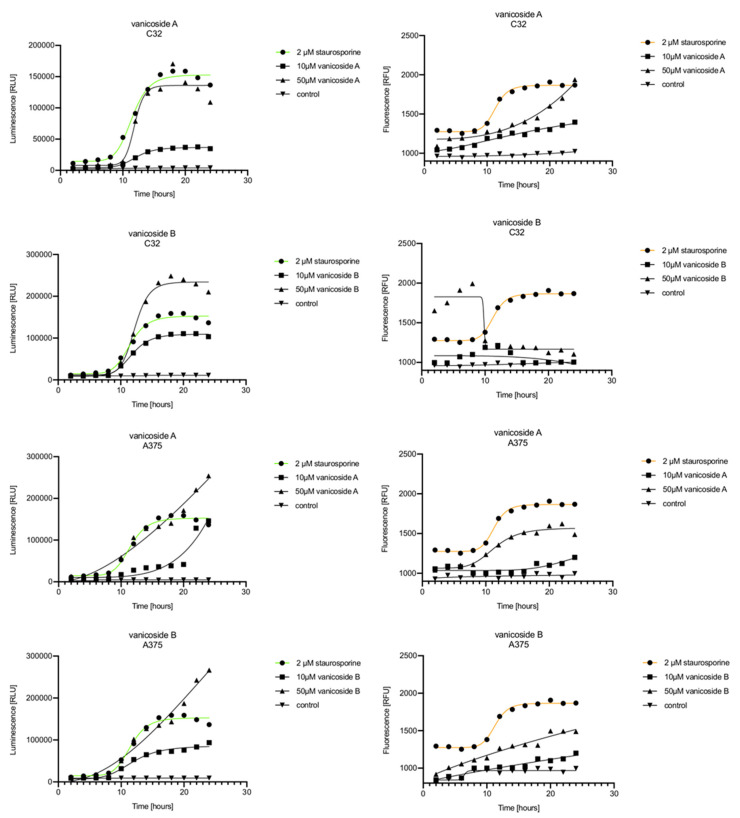
Real-time monitoring of luminescence (A) and fluorescence (B) signals measured for the C32 and A375 cell lines treated with 10 and 50 µM of vanicoside A and vanicoside B over 24 h, reported in relative luminescence units (RLU) and relative fluorescence units (RFU), respectively. Staurosporine (2 µM) was used as the positive control–an apoptosis-inducer. The term “control” means cell lines without treatment of vanicosides. Negative control for staurosporine was not presented.

**Figure 6:**
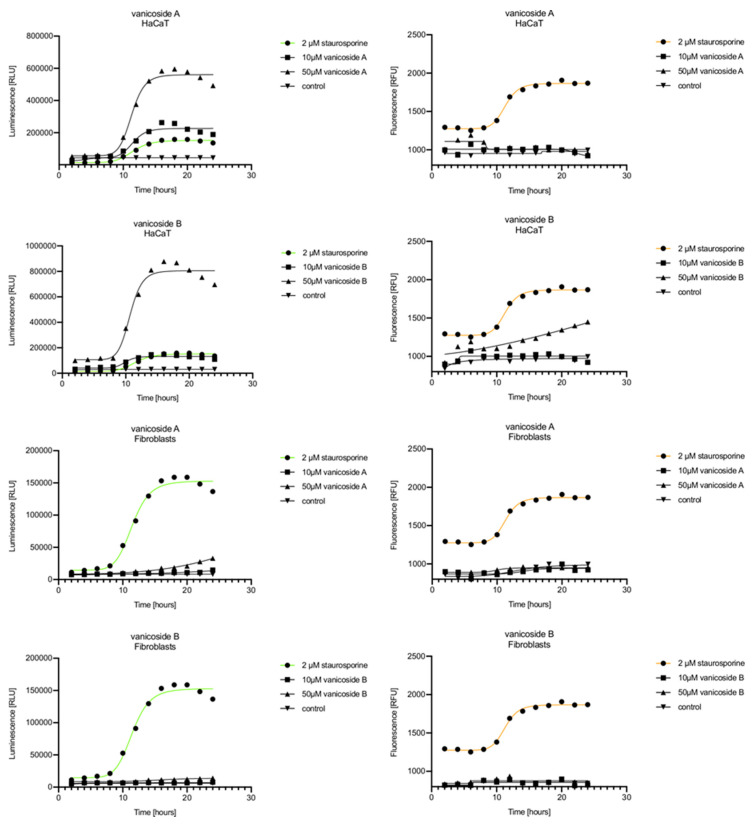
Real-time monitoring of luminescence (A) and fluorescence (B) signals measured for HaCaT and fibroblast cell lines treated with 10 and 50 µM of vanicoside A and vanicoside B over 24 h, reported in relative luminescence units (RLU) and relative fluorescence units (RFU), respectively. Staurosporine (2 µM) was used as the positive control–an apoptosis-inducer. The term “control” means cell lines without treatment of vanicosides. Negative control for staurosporine was not presented.

In the RealTime-Glo™ Annexin V Apoptosis and Necrosis assay, compounds that induce apoptosis (such as staurosporine used in our experiment as the positive control) will produce time- and dose-dependent increases in luminescence—annexin V fusion protein binding to exposed phosphatidylserine (PS) which precede temporal increases in fluorescence due to secondary necrosis (loss of membrane integrity). In this experiment, we have analyzed the response of the C32, A375, HaCaT and fibroblast cell lines treated with 10, 25 and 50 µM of vanicosides A and B over 24 h (Figure 3, Figure 4, Figure 5 and Figure 6).

Vanicoside A initiated a negligible luminescence response from phosphatidylserine (PS) exposure in C32 cells beginning at 2 h at the highest tested concentration (50 µM) and at 4 h for lower concentrations (Figure 3), but a considerable increase in luminescence began between 8 and 10 h of incubation (Figure 5). The luminescence signal grew steadily until 16–18 h. The necrosis detection reagent indicated negligible cell death after 4 h exposure to 50 µM of vanicoside A and the fluorescence signal remained rather constant until 12 h—after that, a slight increase was noted and after 18 h, the increase was considerable and grew steadily until 24 h. For lower doses of vanicoside A (10 and 25 µM), a T-jump in fluorescence increase (compared with the negative control) was noted at 10 h and the next signal grew steadily until 24 h. The luminescence signal was much weaker for 10 and 25 µM of vanicoside A than for 50 µM, which was similar to the luminescence signal for 2 µM of staurosporine (the positive control). The fluorescence signal for 50 µM was similar to that for 10 and 25 µM until 18 h of incubation: after that time, it increased much more than at lower doses. The fluorescence signal curves differed from those typical of staurosporine. A kinetic lag between the significant increase in luminescence and increase in fluorescence was noted, which is consistent with the progression of apoptosis. Moreover, the initial and relatively high level of fluorescence compared with the negative control may indicate an overlap of other types of cell death.

After exposure of the C32 cell line to vanicoside B, a marked enhancement in luminescence was observed between 8 and 10 h and was maximized at 16 h. The luminescence signal was even stronger for vanicoside B than for vanicoside A, but the fluorescence signal was significantly different. For high doses of vanicoside B (25 and 50 µM), the high level of fluorescence was visible from the first measurement, 2 h, and there was no decrease until 10 h, reaching the level close to the control value after 24 h. For the low dose (10 µM), the fluorescence signal grew slightly from 2 h until 10–14 h: after that time, it decreased to the control value. Although the annexin V fusion protein binds to exposed phosphatidylserine, no increase in fluorescence due to secondary necrosis was observed during 24 h incubation.

Exposure of the A375 cell line to 50 µM of vanicoside A caused a slight increase in the magnitude of the luminescence signal after 2 h, a noticeable increase after 6 h and a considerable increase after 8 h. The luminescence signal grew steadily until 24 h. For lower doses of vanicoside A (10 and 25 µM), the luminescence signal was much weaker and noticeably increased after 8 h, but a very significant increase was observed at 20 h. The increase in fluorescence response was observed beginning at 10 h for the 25 and 50 µM doses of vanicoside A and was maximized at 22 h. In addition, 10 µM of vanicoside A caused a weak swing in fluorescence throughout the measurement period and slightly increased at the end, after 24 h incubation. Exposure of the A375 cell line to vanicoside B caused a rather similar luminescence and fluorescence response as vanicoside A (considering fluctuations in the fluorescence signal in the control group). For vanicosides A and B, a kinetic lag between the significant increase in luminescence and increase in fluorescence was found, which indicates the induction of apoptosis.

Exposure of the HaCaT cell line to vanicoside A caused a significant increase in luminescence response beginning at 10h and the signal grew until 16 h, when it achieved the highest level. The luminescence signal was stronger than for the positive control. Vanicoside A did not cause any marked enhancement in fluorescence. Some slight increases in the fluorescence signal were found at the first hours of incubation, but then it decreased to the negative control value. Exposure of the HaCaT cell line to vanicoside B caused a similar luminescence response to vanicoside A, but the signal was significantly stronger at higher concentrations (25 and 50 µM). Differences in the fluorescence level for vanicosides were observed at the highest measured concentration of vanicoside B, 50 µM, where the magnitude of the signal grew steadily until 24 h. For both vanicosides, no increase in fluorescence due to secondary necrosis was observed during 24 h incubation, except for 50 µM of vanicoside B.

There was no significant increase in the luminescence after fibroblast exposure to vanicosides A and B. At the highest concentrations tested, a slight increase in the signal was observed, but it was much lower than for the other tested cell lines (see the luminescence signal scales). No increase in fluorescence was observed.

### 2.3. Molecular Docking Studies

**Figure 7:**
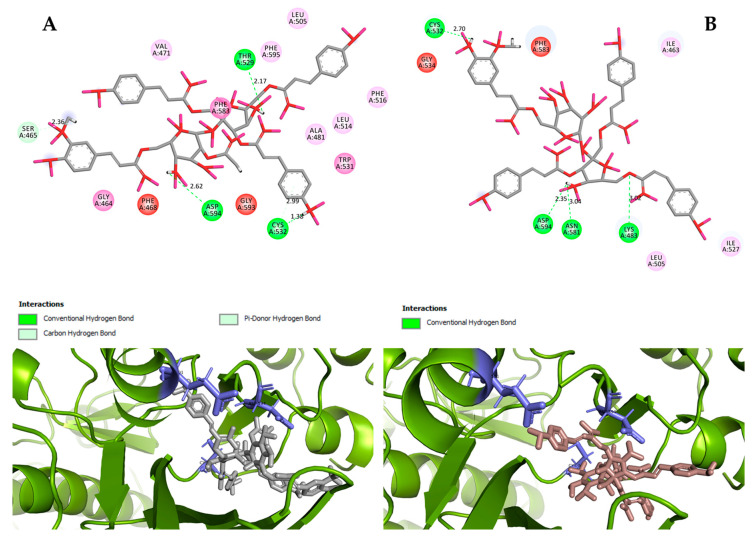
2D and 3D interactions of vanicoside A (**A**) and vanicoside B (**B**) with the B-Raf kinase.

**Figure 8:**
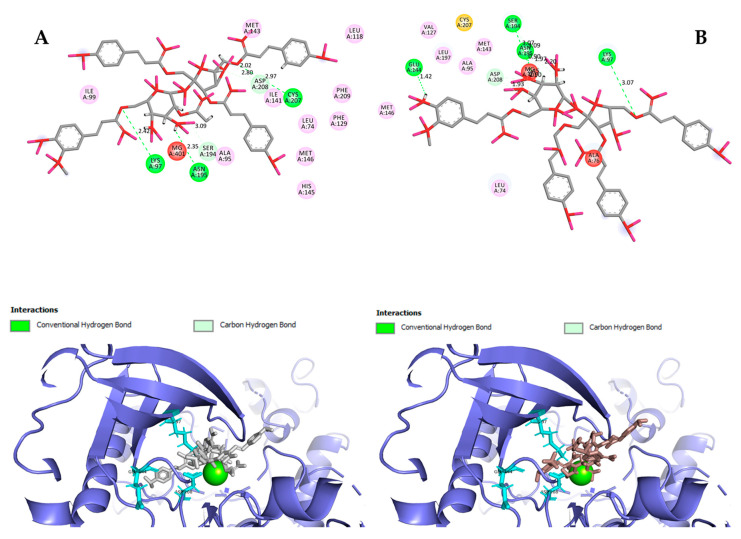
2D and 3D interactions of vanicoside A (**A**) and vanicoside B (**B**) with the MEK-1 kinase.

**Figure 9:**
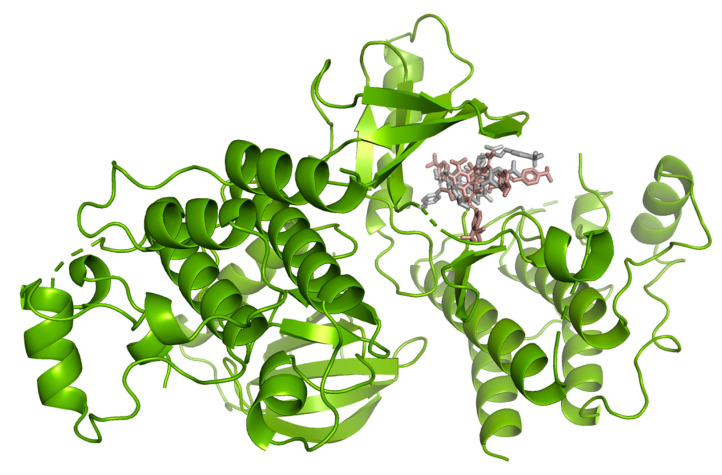
B-Raf kinase (PDB ID: 3OG7) in complex with vanicosides A (gray) and B (pink).

**Figure 10:**
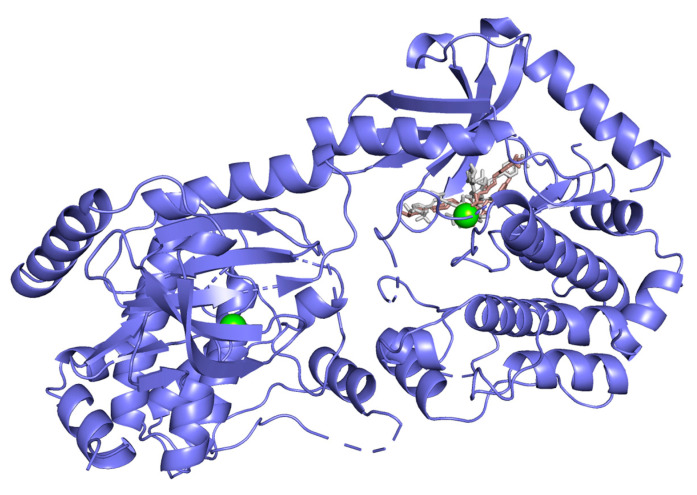
MEK-1 kinase (PDB ID: 3SLS) in complex with vanicoside A (gray) and B (pink).

Hydrogen bond interactions observed in the cases of vanicosides A and B with BRAF(V600E) and MEK1 are presented in the table (Table 1) and compared with interactions of those kinases and their original ligands (known inhibitors of those targets). Additionally, 2D and 3D visualizations of those interactions are provided (Figure 7A,B and Figure 8A,B) as well as visualizations of the whole protein–ligand complexes (Figure 9 and Figure 10).

## 3. Discussion

The cytotoxicity of vanicosides A and B towards melanoma cell lines has not been studied so far; however, the cytotoxicity of other disaccharide esters of phenylpropanoids against different cancer cell lines has been described [14,15]. The results from the MTT assay showed a decrease in the viability of melanoma cells after treatment with vanicosides A and B. Vanicoside A decreased viability of the amelanotic malignant melanoma C32 cell line significantly stronger than vanicoside B, but both vanicosides showed a similar effect against the less sensitive A375 line. The differences observed in the cytotoxicity of vanicosides could result from their different chemical structure and from the differences in the sensitivity of the tested cell lines. The stronger cytotoxic activity of vanicoside A against the C32 cell line can be attributed to an additional acetyl group in vanicoside A. The feruloyl and acetyl groups play an important role in mediating the cytotoxicity of different disaccharide phenylpropanoid esters, which seems to be related to the substitution position of feruloyl groups, where the feruloyl groups at C-6 or C-1′ are critical for cytotoxicity and the increased number of acetyl groups could induce higher tumoricidal activity [14]. The results of our research confirm these observations. Vanicosides A and B have the feruloyl group at C-6 and vanicoside A additionally possesses the acetyl group. Moreover, according to the earlier research, cytotoxicity was weak when C-6 or C-1′ was substituted by the coumaroyl or acetoxyl groups [14,15]. These effects can be explained with a higher redox activity of ferulic acid than of coumaric acid. In compounds with one hydroxyl group, the presence of one or two methoxy groups in the ring additionally increases the antioxidant capacity. It is proved that the methoxy group as an electron-donating group enhances the stabilization of electrons after the transfer of hydrogen from the hydroxyl group to a free radical [23]. In addition, it should be noted that ferulic acid, despite being a strong antioxidant, may generate ROS through the activation of NADPH oxidase, which induces apoptotic cell death in HepG2 human hepatoma cells [24]. An additional confirmation that the position of the feruloyl group is crucial for cytotoxicity is the study by Li et al. [25], in which the isolated new compound named vanicoside A′—being an isomer of vanicoside A with a different link position for the feruloyl moiety—did not show cytotoxicity against four human cancer cell lines, including malignant melanoma cell lines: SK-MEL (malignant melanoma), KB (oral epidermal carcinoma), BT-549 (breast ductal carcinoma) and SK-OV-3 (ovary carcinoma) [25]. Furthermore, studies concerning the antioxidant activity of phenylpropanoid disaccharide esters showed that the increased number of phenolic hydroxyl groups and acetyl groups could produce higher antioxidant activity, and oligosaccharide esters with feruloyl groups exhibited better antioxidant activities than those with coumaroyl groups [14]. By assuming that vanicoside A, with an additional acetyl group, exhibits greater antioxidant activity than vanicoside B (or rather generates more ROS in the melanoma cells), we can explain its stronger cytotoxic effect against the amelanotic line. The question still remains how to explain the different cytotoxic effect of vanicoside A against the A375 cell line and amelanotic C32 cell line. Basically, C32 cell lines and A375 cell lines differ in their melanin content. Melanin plays an important protective role against damage caused by free radicals in melanocytes. Cells with a low melanin content were significantly less resistant to ROS damage than melanocytes with high melanin [26]. Moreover, Hadjur et al. [27] demonstrated that amelanotic melanoma tumor cells were more sensitive to damage by hypericin photosensitization than pigmented cells. Their results revealed that in the absence of melanin, hypericin photosensitization led to generating free radicals (O·^−^_2_, ^•^OH) in melanocytes that affected the metalloenzymes and decreased the intracellular glutathione (GSH) levels. The weakened antioxidant protection and redox imbalance caused cell damage and death. In the presence of melanin, the resistance of pigmented cells was directly related to the protection mediated by the antioxidant enzyme status (catalase, glutathione peroxidase and superoxide dismutase) [27]. Our results from the RealTime-Glo™ Annexin V Apoptosis and Necrosis assay also indicated that in tested melanoma cell lines, the oxidative death mechanism could be involved.

Briefly, in this assay, compounds that induce apoptosis will produce time- and dose-dependent increases in luminescence—annexin V fusion protein binding to exposed phosphatidylserine (PS)], which precede temporal increases in fluorescence due to secondary necrosis (loss of membrane integrity). This kinetic difference in the emergence of the signals is the hallmark of an apoptotic phenotype [28]. Our results from this assay indicated that the apoptotic phenotype of cell death is characteristic of the malignant melanoma A375 cell line, treated with both vanicosides. The response of C32 cells was different for vanicosides A and B. While the vanicoside A caused a considerable increase in luminescence and (characteristic of secondary necrosis lag) increase in fluorescence, vanicoside B—despite causing an increase in luminescence—did not show any increase in fluorescence through 24 h of incubation. Moreover, both vanicosides caused the initial, relatively high level of fluorescence compared with the negative control. This initial high level of fluorescence was especially visible to vanicoside B, where the signal decreased to the control value through 24 h of incubation. The differences between vanicosides A and B in the necrosis detection reagent observed in the annexin assay are reflected in the results from the MTT assay, where vanicoside B did not show any clear cytotoxicity towards the C-32 cell line during 24 h incubation. Nevertheless, during longer incubation (i.e., after 48 and 72 h), the cytotoxic effect of vanicoside B was significant; unfortunately, we collected date from the fluorescence response only regarding 24 h incubation. We can assume that after longer incubation of C32 cells with vanicoside B (48, 72 h), an increase in membrane permeability could be observed, but further research is needed. In our view, the initial loss of membrane integrity (slight for vanicoside A and significant for vanicoside B) indicates a more complex response of melanoma cells for vanicosides than only induction of apoptosis: an overlap of different types of cell death could occur. It should be mentioned that in some cases, such as oxygen-radical injury of cells, two cell death mechanisms—apoptosis and permeability transition pore (PTP)-mediated necrosis—occur simultaneously [29]. Vanicosides, like other polyphenols, could promote oxidative stress in cancer cells and lead to cell death simultaneously through various mechanisms [7]. Research showing the effect of vanicosides on mitochondrial membrane permeability, in particular, on mitochondrial outer membrane permeabilization (MOMP: plays an essential role in apoptosis) and permeability transition pore (PTP; associated with necrosis), is recommended in the future [29]. Importantly, taking into account that vanicoside A and B could harm melanoma cell lines by the oxidative death mechanism, it could be supposed that they could also influence non-mutated cell lines (with the wild-type of the *BRAF* gene) which significantly expands their potential therapeutic range.

However, the results of our study enable us to suppose that both vanicosides could affect the viability of malignant melanoma cells by inhibiting the BRAF(V600E) and MEK1 kinases. Molecular docking analyses show that both vanicosides were successfully docked into the active sites of the BRAF(V600E) and MEK1 kinases. Vanicosides A and B form four hydrogen bonds with BRAF kinase residues. The interaction with the Asp594 residue is present among them and this is a crucial interaction in the case of the original PLX4032 ligand (ligand of 3OG7 protein structure) [30]. Lys483 and Cys532 residues are also reported as important residues for the binding of BRAF potential inhibitors [31,32]. Vanicoside B interacts with both of them (Figure 7B), whereas vanicoside A with Lys483 only (Figure 7A). In the case of MEK1, both vanicosides form five hydrogen bonds with the residues of this kinase (Figure 8A,B). Interaction with the Lys97 residue, which is an important residue for the catalytic activity of MEK-1, is observed in the case of both vanicosides, as well as interaction with Asp208 residues that is also present in the case of the original ligand UCB1353770 (ligand of 3SLS protein structure) [33,34,35]. Those results suggest that both vanicosides could be considered as potential inhibitors of those targets as well as further tested experimentally.

There is only one more study that showed the effect of vanicosides A and B on cancer-related kinase activity [16]. Zimmerman et al. revealed that vanicosides A and B inhibited protein kinase C (PKC) activity at IC_50_ values of 44 and 31 μg/ml, respectively. In this study, both vanicosides also showed cytotoxicity against the MCF cell line at submicromolar dose levels [16]. Unfortunately, studies on the modulation of PKC activity by vanicosides are not well-documented. Since there is a correlation between abnormalities in PKC signaling and cancer, a detailed study concerning the influence of vanicosides on protein kinase C should be conducted in the future [36]. It should be mentioned that staurosporine, used in our study as an apoptosis-inducer (the positive control), is an established inhibitor of protein kinase C through the prevention of ATP binding to the kinase [36].

The dose-dependent mechanism of cell death should be discussed as well. Interestingly, treatment of C32 cells with doses between 5–50 µM of vanicoside A decreases cell viability to some similar level and the next significant decrease was noted after treatment with 100 µM of vanicoside A. The results from the annexin assay are also very similar for lower concentrations of vanicosides. Different doses could reflect different mechanisms of cell death, which is observed in studies with polyphenols, where high doses of drugs promote apoptosis, but low concentrations of drugs exert a pro-senescence effect [7].

Another issue that needs to be resolved in the future is the impact of vanicosides on the GLUT protein. This is important because of the presence of a glucose molecule in the chemical structure of vanicosides and because the earlier studies revealed increased GLUT1 (glucose transporter type 1) expression in malignant melanoma, which promotes glucose uptake and cell growth [37]. Increased transport of vanicoside A to the melanoma cells via GLUT1 transporters or, on the contrary, stronger blocking of GLUT1 by vanicoside A may affect its stronger cytotoxic effect than vanicoside B, observed in our research.

To check whether vanicosides are selectively cytotoxic against the melanoma cell lines, we performed a cell viability assay (MTT) and RealTime-Glo™ Annexin V Apoptosis and Necrosis assay on two normal cell lines—keratinocytes (HaCaT) and the primary fibroblast line. The viability of normal cell lines—keratinocytes (HaCaT) and the primary fibroblast line—was decreased clearly only after treatment with high concentrations of vanicosides—25 or 50 µM (depending on the vanicoside). The HaCaT line was more sensitive to vanicoside A than vanicoside B. The toxic effect of vanicoside A against keratinocytes (HaCaT) manifested at 25 µM and increased with the concentration of vanicoside A, but not with incubation time (about 60% cell viability for all incubation times). Vanicoside A at 25 µM caused similar decreased cell viability of the HaCaT, C32 and A 375 lines after 72 h. An even stronger decreased cell viability of HaCaT than melanoma lines was observed after 24 and 48 h incubation. However, what is important, incubation of HaCaT with low concentrations of vanicoside A (2.5–10.0 µM), where the toxic effect against the C 32 line was significant, did not clearly cause a decrease in the cell viability of keratinocytes (cell viability slightly decreased after 24 and 48 h incubation, but then after 72 h, increased to 102%—10.0 µM). Further, the A375 line viability was significant more affected at low concentrations of vanicoside A than HaCaT, after 72 h. These results could reflect different mechanisms leading to decreased cell viability between the HaCaT and melanoma cell lines. The fibroblasts appeared to be more resistant for both vanicosides than keratinocytes. Clear differences between HaCaT and fibroblast were seen in the results from the annexin V assay. Incubation of the HaCaT line with vanicosides caused a strong luminescence signal, while there was no significant increase in the luminescence and fluorescence signal after fibroblast exposure to vanicosides. However, for both vanicosides, despite the annexin V fusion protein binds to exposed phosphatidylserine in the HaCaT line, the increases in fluorescence due to secondary necrosis were not observed during 24 h incubation, except for 50 µM of vanicoside B (characterized with the highest level of luminescence, higher than for 2 µM of staurosporine and slight increase in fluorescence). In the case where a decrease in the viability of HaCaT and fibroblasts was observed, but without signs of secondary necrosis, we have concluded that mitochondrial activity could have been decreased instead of leading to cell death. It is worth noting that MTT reduction is a marker reflecting viable cell metabolism, not specifically cell proliferation [38].

It is also worth noting that both keratinocytes and fibroblasts chosen for the study showed a proliferation rate similar to the tested melanoma cells. For the subculture, we used 2.5 × 10^4^ cells per T75 cell culture flask for each of the tested cell lines and we obtained bottle confluency after three days of culture. Human fibroblasts have high proliferative potential and similar results were obtained by Boukamp et al. [39]. In turn, the HaCaT cell line is a spontaneously immortalized aneuploid human keratinocyte cell line, hence the proliferative potential of these cells is similar to the other tested cell lines [40]. For this reason, we suspect that the differences in the response of the tested cells to the tested vanicosides are rather due to the differences in the metabolism of these substances by cancer cells and normal cells [41,42]. These differences may also be the result of the varied uptake rate or availability of cellular targets. These hypotheses must be, however, further explored using a high-content analysis approach which is beyond the scope of the present study. For such an experimental approach, additionally stimulated by the results of molecular docking, much higher amounts of the selected bioactive compounds will have to be isolated [43].

## 4. Materials and Methods

### 4.1. Isolation of Vanicosides

Vanicoside A and vanicoside B were isolated from the rhizomes of *Reynoutria sachalinensis* [F.Schmidt] Nakai, and identified according to the procedure described in our previous paper [11].

### 4.2. Cell Culture

Four human cell lines were tested: two melanoma cell lines - the amelanotic malignant melanoma C32(BRAFV600E) and the malignant melanoma A375(BRAFV600E) as well as two normal cell lines—keratinocytes (HaCaT) and the primary fibroblast line—. The C32, A375 and HaCaT cell lines were purchased from ATCC^®^ (LGC Standards, Łomianki, Poland). Human fibroblasts were taken with the patient’s consent from the oral cavity. The cells were grown as a monolayer in Dulbecco modified Eagle medium (DMEM, Sigma-Aldrich, St. Louis, MO, USA), which was supplemented by 2 mM L-glutamine, 10% fetal bovine serum (FBS, Sigma-Aldrich, Poland) and 50 μg/ml streptomycin (Sigma-Aldrich, Poland) at 37 °C, 5% CO_2_. Before every experiment, cells were removed by 0.25% trypsin with 0.02% EDTA (Sigma-Aldrich, St. Louis, MO, USA).

### 4.3. Cell Viability–MTT Assay

The viability of cells was determined by an MTT assay (Sigma-Aldrich, St. Louis, MO, USA) after experiments with different concentrations of vanicoside A or vanicoside B. Dimethyl sulfoxide (DMSO, Sigma-Aldrich, St. Louis, MO, USA) was used as a solvent for vanicosides. The following concentrations were used for the experiment: 2.5, 5, 10, 25, 50 and 100 µM. The MTT assay was used to estimate a mitochondrial metabolic function through the measurement of mitochondrial dehydrogenase after 24, 48 and 72 h incubation after experiments. For the experiment, the cells were seeded in 96-well microculture plates at 1 × 10^4^ cells/well. After the incubation with selected concentrations of vanicoside A or B, the experiments were conducted according to the manufacturer’s protocol. The absorbance was determined using a multi-well scanning spectrophotometer at 570 nm (GloMax^®^ Discover Microplate Reader, Promega, Madison, WI, USA). The mitochondrial metabolic function was expressed as a percentage of viable treated cells in relation to untreated control cells. Staurosporine was used as the cytotoxic positive control at 2 μM.

### 4.4. RealTime-Glo™ Annexin V Apoptosis and Necrosis Assay

Cells were seeded in 50 µL growth medium in white 96-well microtiter plates at the concentration of 1 × 10^4^ cells/well. A set of control wells without cells present (growth medium only) to determine background luminescence and background fluorescence was included. The appropriate dilution of vanicoside A (or B) was prepared in growth medium at 4× the desired final concentration. A set of control wells without a vanicoside present (untreated controls) was included. An amount of 50 µL of the vanicoside A (or B) dilution was added to the appropriate wells in the 96-well assay plate. Then, 100 µL of 2× concentrated RealTime-Glo™ Annexin V Apoptosis and Necrosis Detection Reagent in growth medium was added to each well. Cells were incubated in a covered 96-well assay plate at 37 °C/5% CO_2_ in a humidified cell culture incubator. Luminescence and fluorescence (Ex 475, Em 500–550) on the GloMax^®^ Discover instrument (Promega, Madison, WI, USA) using the RealTimeGlo™ Annexin V Apoptosis Assay pre-programmed protocol at 2, 4, 6, 8, 10, 12, 14, 16, 18, 20, 22 and 24 h were measured.

### 4.5. Molecular Docking Studies

For the molecular docking studies, two melanoma targets were selected: B-Raf kinase V600E oncogenic mutant and MEK-1 kinase. Docking was performed with the GOLD (version 5.7.2, Cambridge Crystallographic Data Centre, Cambridge, UK) software [44] and crystal structures of target proteins were obtained from the PDB database: BRAF (V600E) kinase with PDB ID:3OG7 and MEK-1 kinase with PDB ID:3SLS. Ligands of those proteins and water molecules were removed from the PDB files and hydrogens were added.

The binding site was determined based on original ligand interactions in the active site and residues within 10 Å from the original ligand. The number of poses to be generated was set to 10 and GoldScore was used as the scoring function to rank the docked compounds. The final protocol for the docking was based on successful re-docking of the reported ligands. Docking results were analyzed with the Accelrys Discovery Studio software 4.1. (San Diego, CA, USA) [45] and best docking poses were selected on the basis of visual inspections of the receptor–ligand interactions and docking scores. The Discovery Studios software was also used to generate 2D graphical representations of the docking results. The representations of the docking results in 3D were done with PYMOL (version 2.3.5, Molecular Graphics System, Schrödinger LLC, New York, NY, USA) [46].

### 4.6. Statistical Analysis

All the experimental procedures were performed in triplicate. The values obtained from the MTT and RealTime-Glo™ Annexin V Apoptosis and Necrosis assay are presented as the mean of three replicates ± SD. To evaluate the distribution of the results, the Shapiro–Wilk test was used. The statistical analysis was performed by Student’s *t*-test using GraphPad Prism v. 7 (GraphPad Software, San Diego, CA, USA). The *t*-test was used for the means comparison between treatments and the control (cells without vanicosides) samples. *p* ≤ 0.05 or *p* ≤ 0.0001 were considered statistically significant. The graph of time- and dose-dependent increases in luminescence and fluorescence was presented using the non-linear regression fit (least squares fit) of GraphPad Prism v. 7.

## 5. Conclusions

In conclusion, both tested phenylpropanoid esters from *R. sachalinensis* demonstrated significant cytotoxicity against amelanotic and melanotic melanoma cell lines bearing the BRAFV600E mutation. Vanicoside A decreased the viability of the amelanotic C32 cell line significantly stronger than vanicoside B, whereas both compounds showed similar effect against the less sensitive A375 cell line. The observed differences in cytotoxicity could result from the additional acetyl group in vanicoside A, and from the differences in the tested melanoma cell lines—lower melanin content in amelanotic cells, suggesting that in the melanoma cell lines, an oxidative stress-related death mechanism could be involved. Moreover, vanicosides caused the death of melanoma cells at concentrations from 2.5 to 50 µM, without harming the primary fibroblast line. The keratinocyte cell line (HaCaT) was more sensitive to vanicosides than fibroblasts, showing a clear decrease in viability after incubation with 25 µM of vanicoside A as well significant phosphatidylserine (PS) exposure, but without a measurable cell death-related fluorescence. Therefore, further studies are warranted to ensure the selectivity against cancerous cells and safety. Vanicosides A and B induced an apoptotic death pathway in the melanoma cell lines. However, due to the initial loss of cell membrane integrity, an additional cell death mechanism might be involved like PTP-mediated necrosis. Molecular docking revealed that both vanicosides may be able to bind to the active sites of the B-RafV600E and MEK-1 kinases. The resulting kinase inhibition could interfere with the RAF-MEK-ERK signaling cascade during melanocytic neoplasia, but this finding must be verified using a cell model.

## Figures and Tables

**Table 1:** Hydrogen bond interactions of vanicosides A and B with the BRAF(V600E) and MEK1 kinases.

Target	Ligand Interactions	Vanicoside A	Vanicoside B
BRAF(V600E) kinase (ligand: PLX4032)	Asp594, Phe595, Trp531	Asp594, Cys532, Thr529, Ser465	Asp594, Cys532, Lys483, Asn581
MEK-1 kinase (ligand: UCB135377)	Lys97, Asp208, Val211, Ser212,	Lys97, Asp208, Ser194, Asn195, Cys207	Lys97, Asp208, Ser194, Asn195, Glu144
